# Complex peptide macrocycle optimization: combining NMR restraints with conformational analysis to guide structure-based and ligand-based design

**DOI:** 10.1007/s10822-023-00524-2

**Published:** 2023-08-03

**Authors:** Ajay N. Jain, Alexander C. Brueckner, Christine Jorge, Ann E. Cleves, Purnima Khandelwal, Janet Caceres Cortes, Luciano Mueller

**Affiliations:** 1 Research and Development, BioPharmics LLC, Sonoma County, CA USA; 2 Bristol-Myers Squibb Company, Princeton, NJ USA

**Keywords:** PD-L1, Macrocycle, NMR, ForceGen, Surflex-Dock, eSim, Ligand-strain

## Abstract

Systematic optimization of large macrocyclic peptide ligands is a serious challenge. Here, we describe an approach for lead-optimization using the PD-1/PD-L1 system as a retrospective example of moving from initial lead compound to clinical candidate. We show how conformational restraints can be derived by exploiting NMR data to identify low-energy solution ensembles of a lead compound. Such restraints can be used to focus conformational search for analogs in order to accurately predict bound ligand poses through molecular docking and thereby estimate ligand strain and protein-ligand intermolecular binding energy. We also describe an analogous ligand-based approach that employs molecular similarity optimization to predict bound poses. Both approaches are shown to be effective for prioritizing lead-compound analogs. Surprisingly, relatively small ligand modifications, which may have minimal effects on predicted bound pose or intermolecular interactions, often lead to large changes in estimated strain that have dominating effects on overall binding energy estimates. Effective macrocyclic conformational search is crucial, whether in the context of NMR-based restraints, X-ray ligand refinement, partial torsional restraint for docking/ligand-similarity calculations or agnostic search for nominal global minima. Lead optimization for peptidic macrocycles can be made more productive using a multi-disciplinary approach that combines biophysical data with practical and efficient computational methods.

## Introduction

Affinity-based selection of in vitro expressed macrocyclic peptides using modern mRNA-display technology can identify relatively potent and selective lead compounds [1]. However, systematic optimization of large macrocyclic peptide ligands is a serious challenge. Here, we describe an approach for optimization of such leads using the PD-1/PD-L1 system as a retrospective example of moving from initial lead compound to clinical candidate. We show how conformational restraints can be derived by exploiting NMR data to identify low-energy solution ensembles of a lead compound.

**Fig. 1:**
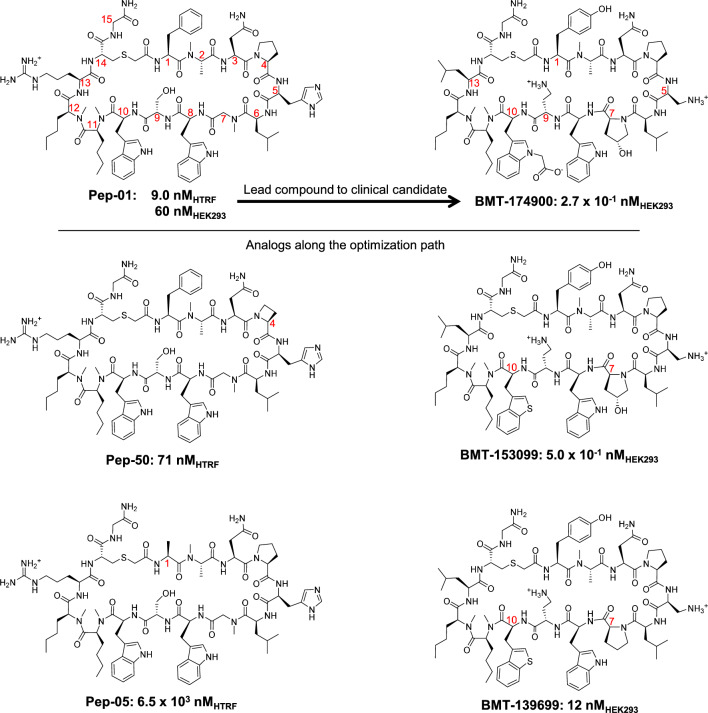
Examples of macrocyclic PD-1/PD-L1 antagonists: three examples from a patent disclosure with IC_50_ values for human PD-L1/PD-1 binding (left column), measured by homogeneous time resolved fluorescence (HTRF) and three examples from the subsequent lead optimization effort (right column) with IC_50_ values measuring inhibition of soluble PD-1 binding to PD-L1 expressed on the surface of HEK293 cells

A PD-L1 lead compound and numerous analogs were disclosed in a patent filing that became public in 2016 [2] that demonstrated both efficacious ligand-binding and blockade of the interaction between PD-L1 and PD-1. Figure 1 (left column) shows three examples from the initial disclosure in decreasing order of potency along with three examples from the lead optimization effort (right column). BMT-174900 (also known as BMS-986189) is currently in human clinical trials along with a number of other candidates targeting the PD-1/PD-L1 interface for cancer therapies [3]. In moving from the initial lead compound to the clinical candidate, modifications to 6 positions in the macrocyclic peptide were required. This was accomplished through structure-based drug design, in an iterative process that required synthesis and evaluation of thousands of compounds. The process was guided by multiple co-crystal structures of macrocyclic ligands with PD-L1, but the path to BMT-174900 did not make extensive use of the types of computational approaches in common use on smaller non-macrocyclic molecules.

The example structures in Fig. 1 exhibit high sensitivity to minor structural changes. The change from Pep-01 to Pep-50 involves the deletion of a single methylene at position 4, changing a proline into the corresponding azetidine non-natural amino acid, resulting in a decrease of nearly a log unit of pIC_50_. Similarly, the change from Phe in Pep-01 to Ala in Pep-05 at position 1 yielded a decrease of nearly 3 log units. As we shall see, these dramatic shifts in activity can be only partially explained by protein-ligand binding interactions, with changes in conformational energetics playing a crucial role. The changes required to move from lead-compound Pep-01 to clinical candidate BMT-174900 took place at 6 positions and included explorations of both natural and non-natural amino acids. Systematic exploration of just five conservative alternatives at each of those 6 positions would require over 7000 analogs, with such systematic exploration at all 15 positions requiring over 750,000 analogs. In this study, we analyze the extent to which recently developed approaches for modeling macrocyclic ligands can be of use in such lead optimization projects going forward.

Over the past several years, methods for computational modeling of macrocyclic ligands have made significant progress [4–9]. In particular, natural-product based and semi-synthetic macrocycles of up to roughly 21–23 total rotatable bonds (including both macrocyclic bonds and exocyclic bonds) have been shown to be tractable, in terms of accuracy and speed of conformational search when utilizing multiple computing-cores [9]. However, larger peptidic macrocycles remain challenging, especially in cases where “ladders” of trans-annular hydrogen bonds do not form stabilizing networks. For comparison, the examples shown in Fig. 1 each have 60 or more total rotatable bonds—well beyond the tractable range without biophysical data to reduce the search space. Recently, we have shown how distance and dihedral restraints derived from NMR measurements can be used to elucidate low-energy solution ensembles for peptidic macrocycles [9–11].

**Fig. 2:**
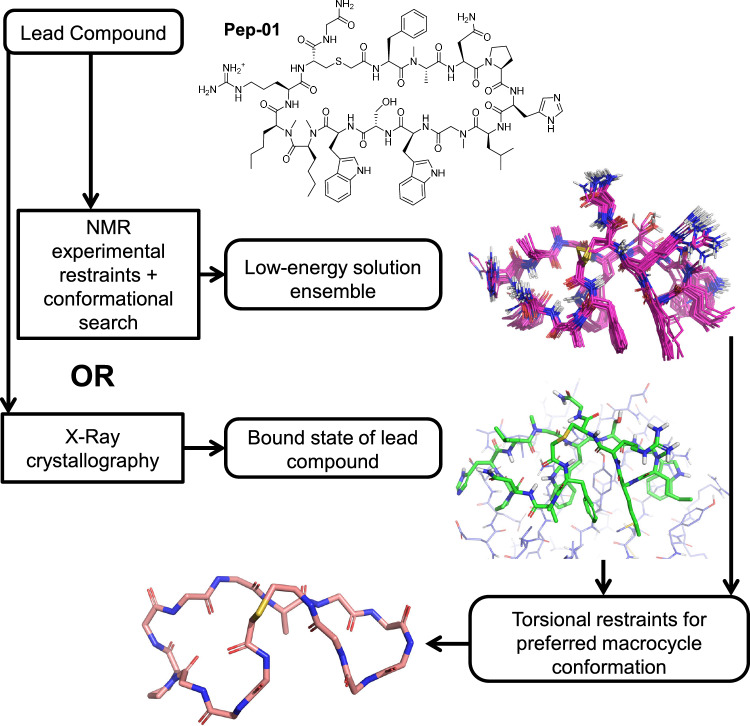
Scheme for deriving a macrocycle conformational preference, either from NMR-restrained conformational search or from X-ray crystallography

Figure 2 illustrates how a preferred macrocycle conformation can be derived from either NMR-restrained conformational search [9] or from X-ray crystallography coupled with careful refinement of the bound macrocycle coordinates [12, 13]. In many cases, obtaining an X-ray co-crystal structure of sufficient quality can be insurmountable. For heavily-selected macrocyclic structures (either through evolutionary pressures for natural products or through screening of very large libraries), the solution state often reflects a large degree of pre-organization toward the bound state. From either a well-fit conformation into X-ray density (green in Fig. 2) or a representative exemplar from a low-energy pool of conformers that satisfy NMR restraints (magenta carbons), a substructure can be used to define a conformational preference. The substructure (salmon carbons) at the bottom of Fig. 2 was extracted from the lowest-energy conformer of the NMR solution ensemble shown in magenta.

**Fig. 3:**
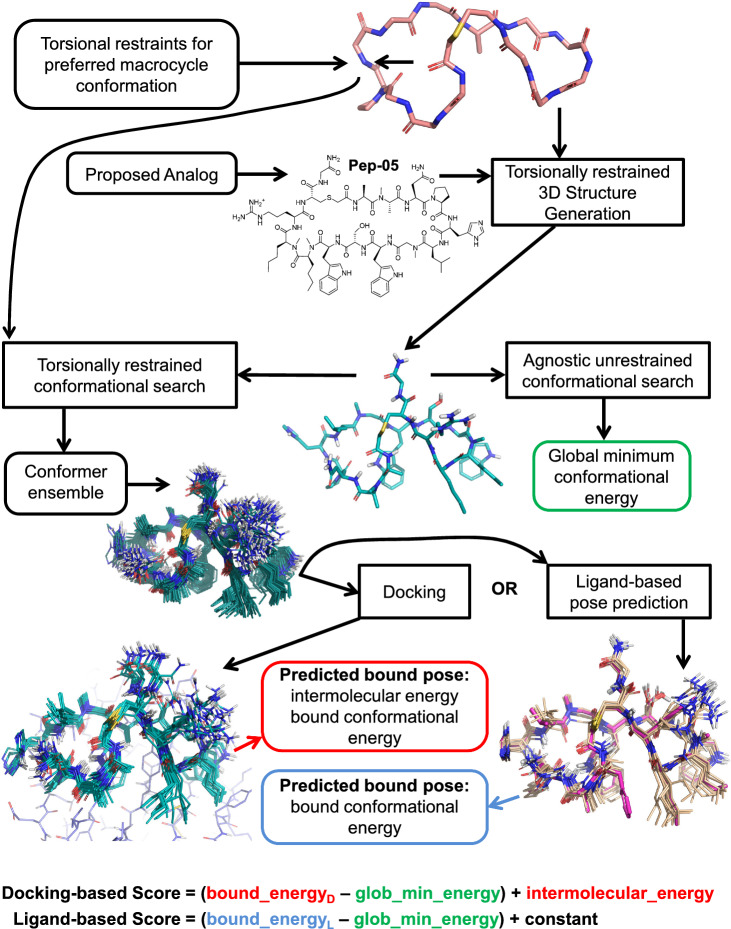
Scheme for exploiting a macrocycle conformational preference to predict a bound pose, either using docking (protein structure shown in slate carbons at bottom left) or ligand similarity (exemplar conformer target shown in magenta carbons at bottom right). For the ligand-based score, a constant value of − 24.0 kcal/mol was added to the estimated strain energy in order to put the scores from the two protocols on the same rough scale

Figure 3 illustrates how the conformational preference can be used to guide conformational search toward predicting the bound state of new analogs. Adherence to that preference can be implemented via graph matching of proposed analogs to the molecular fragment (salmon carbons, top). The subgraph match between a new analog and the given fragment is used to instantiate torsional restraints to match the conformation of the fragment. The restraints are applied through the use of square-welled quadratic energy penalties that allow for zero penalty within some tolerance to deviations from the preferred torsional angle. Structure generation is done with the given restraints and conformational search is done both with and without the restraints. For the parts of the molecule that match the torsional restraint, relatively little conformational variation occurs. For the unmatched parts of the molecule, a great deal of variation may be present, subject to the considerations of energetics. The restrained conformer ensemble is used as input to either molecular docking or molecular similarity calculations to predict the bound pose of the analog (bottom left and right of Fig. 3). The pose optimization that occurs during docking or similarity-based optimization results in well-focused bound conformer ensembles, as seen at the bottom of Fig. 3. The unrestrained ensemble is used to identify the global minimum energy.

Docking, of course, requires at least one example of a compliant protein conformation (bottom left, Fig. 3, in slate carbons). The structure-based protocol produces an intermolecular energy value in addition to a bound conformational energy value. The bound conformational energy together with the global minimum provide an estimate of bound ligand strain (the parenthetical values in score definitions at the bottom of Fig. 3). For the structure-based score, the intermolecular energy is added to the bound ligand strain, resulting in a final estimate for the enthalpic component of the protein-ligand binding energy. The structure-based protocol benefits from the ability to identify new interactions with the protein for well-designed analogs.

For the purely ligand-based protocol, the analog’s restrained conformer ensemble is aligned to an exemplar from the NMR-based solution-ensemble of the lead compound (bottom right, Fig. 3, magenta carbons), making use of the eSim methodology [14]. This similarity-based alignment is used for bound pose prediction, providing an analogous bound conformational energy value to that obtained in the structure-based protocol. Note that the nominal similarity score value may not be of use in compound ranking when seeking significant increases in potency, which requires deviation from the lead compound (leading to lower similarity scores). In a design scenario seeking to maintain potency while diversifying underlying chemical structure, the similarity score values may be of use. But in this work, only the poses that result from the similarity optimization process are used instead of using the similarity score.

Surprisingly, relatively small ligand modifications, which may have minimal effects on the predicted bound pose or intermolecular interactions, often lead to large changes in estimated strain that have dominating effects on overall binding energy estimates. In this work, changes in estimated ligand strain explain the largest fraction of variation in measured activity. The importance of the differences in the energy estimates for bound and solution states places a premium on effective macrocyclic conformational search. Conformational search must be thorough and efficient, whether in the context of NMR-based restraints, X-ray ligand refinement, partial torsional restraints for docking or ligand similarity calculations or agnostic search for nominal global minima.

In what follows, a large set of analogs of the initial lead compound are subjected to the retrospective application of the structure-based and ligand-based workflows just described. While calculations that make use of a protein structure provide more information, a purely ligand-based workflow can be valuable due to the large effects seen from estimates of bound ligand strain. Lead optimization for peptidic macrocycles can be made more productive using a multi-disciplinary approach that combines biophysical data with practical and efficient computational methods.

Data and methods discussed in this paper are available to other researchers (see Declarations).

## Results and discussion

Results for applying two computational workflows for prioritizing analogs of lead-compound Pep-01 will be described: (1) a structure-based method requiring a crystal structure of PD-L1 in a compliant conformation to bind macrocycles in this series and (2) a purely ligand-based method. Both approaches make use of information to partially constrain the conformational space required to be searched to make predictions of bound poses. The information can be derived from experimental NMR data for Pep-01 in its solution state, a co-crystal structure of Pep-01 bound to PD-L1, or a structure-based prediction of the bound state of Pep-01 to a non-cognate protein conformation.

### Correspondence of Pep-01 NMR solution ensemble to its bound state

The NMR experimental analysis of Pep-01 yielded 50 distance restraints between single proton pairs, 115 distance restraints where one/both ends contained chemically equivalent protons and six torsional restraints consisting of 1 omega and 5 psi angles. Very thorough conformational search was performed using the deep ForceGen approach [15]. Refer to the Methods and Data for additional details on the NMR experimental aspects and conformational search methods.

**Fig. 4:**
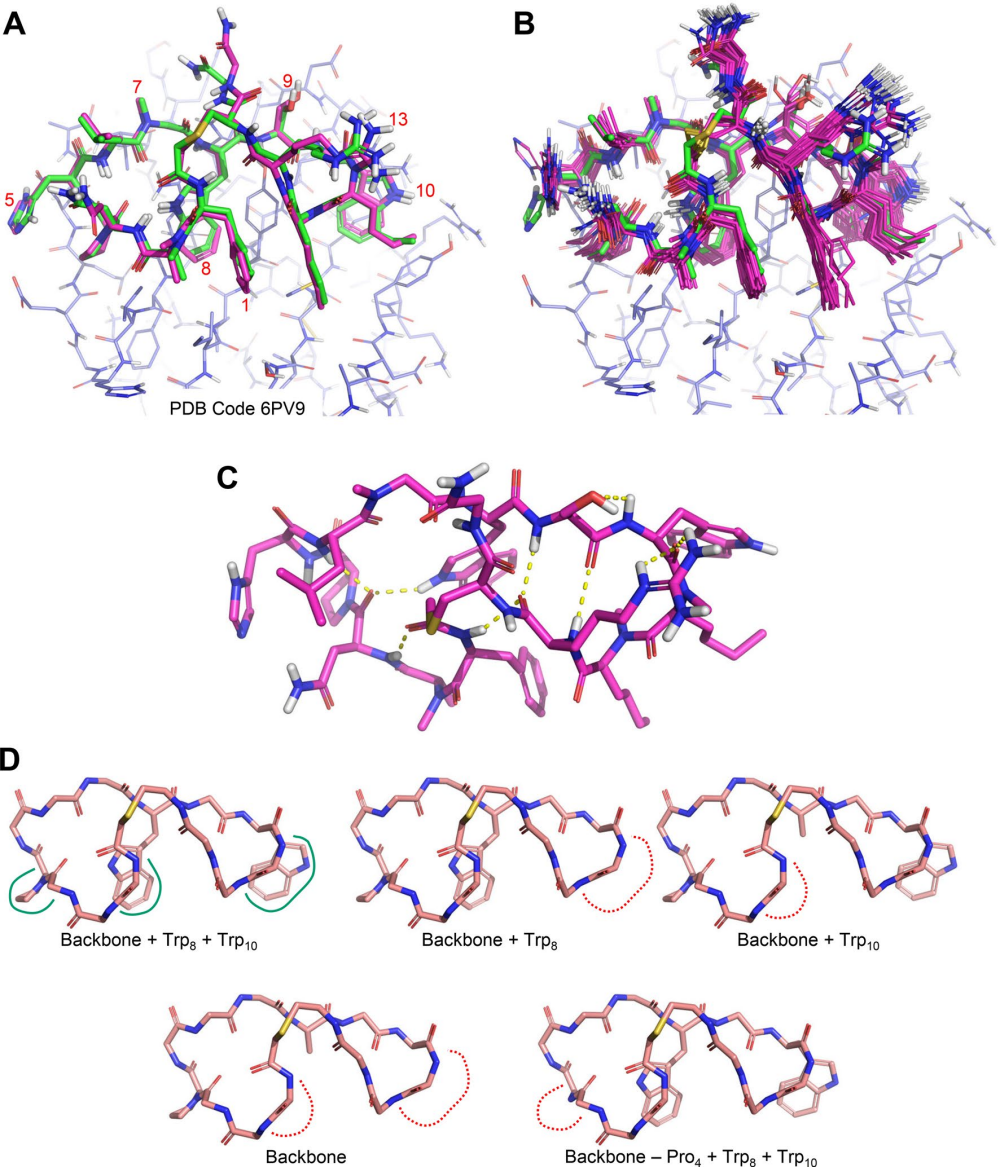
Solution ensemble for Pep-01 from NMR restrained conformational search: **A** the best-matching conformation from the ensemble (magenta carbons) and the bound state by crystallography (green carbons), with sidechains of specific residues numbered in red; **B** the 24 lowest energy non-redundant conformers (within 5 kcal/mol of the minimum, magenta) superimposed on the bound state (green), **C** the single lowest energy conformer (viewed from the solvent-exposed side) with H-bonds labeled; **D** five alternative molecular subfragments derived from the lowest energy conformer

Figure 4A shows the comparison between the PD-L1 bound state of Pep-01 (green carbons) [1, 2] and the closest-matching conformer from the ensemble that came from the NMR-restrained conformational search procedure. The particular conformation shown from the NMR-based ensemble (magenta carbons) was 6 kcal/mol above the lowest-energy example, and it was a very close match to the xGen re-fit bound ligand state (0.9 Å RMSD for all non-hydrogen atoms and 0.4 Å RMSD for ring backbone atoms). Figure 4B shows the set of non-redundant conformers from the lowest 5 kcal/mol energy window. The single lowest energy conformer was 1.4 Å RMSD from the bound state (0.4 Å RMSD for ring backbone atoms).

Clearly, the solution-state of Pep-01 is pre-organized for binding PD-L1. In particular, buried sidechains (residues 8, 1 and 10 especially) showed relatively little movement in the solution ensemble. By contrast, solvent-exposed residues (e.g. 13 and 5) with little protein contact exhibited more movement. Within the backbone itself, there are five hydrogen bonds between amide carbonyl oxygen atoms and amide protons, with an additional one between the indole N-H of Trp_8_ and a backbone carbonyl oxygen (see Fig. 4C). Note that these H-bonds do not form a topologically detectable beta-hairpin-like structure [9] but form a rather unique stabilizing framework.

Figure 4D shows five alternative molecular subfragments derived from the lowest energy conformer of the NMR-restrained solution ensemble. These are used to establish conformational preferences for analogs by employing graph matching. Given an analog, the subfragments are matched in order (left to right, top to bottom), and the first match is used to instantiate a set of torsional preferences for the analog during conformational search (as described earlier in the discussion of Fig. 3). The fragments are ordered from most restrained to least, with the fifth alternative allowing matches to variants at Pro_4_ that retain both Trp residues (of which there are a few among the patent peptides).

**Fig. 5:**
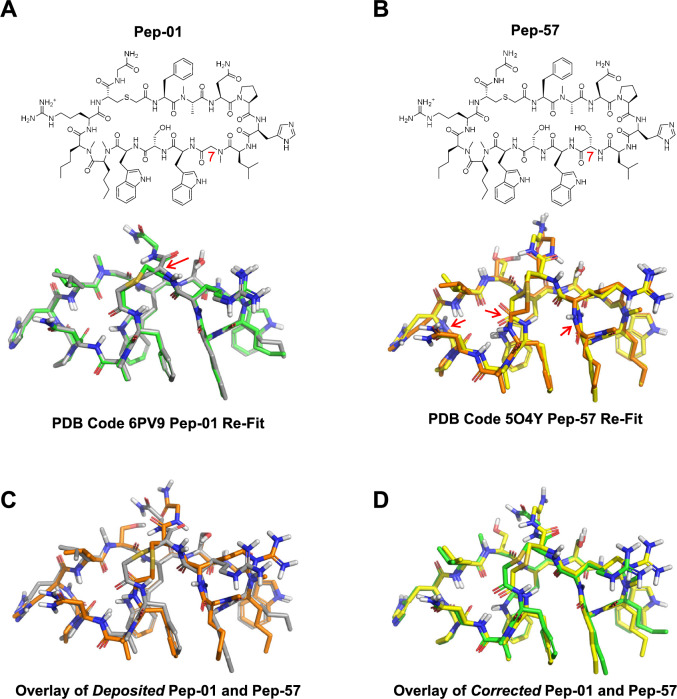
Comparative overlays of different ligand fits to X-ray density: **A** original deposited Pep-01 structure (gray) and corrected real-space fit (green); **B** original deposited Pep-57 structure (orange) and corrected real-space fit (yellow); **C** original deposited Pep-01 (gray) and Pep-57 structures (orange); **D** corrected xGen re-fit Pep-01 (green) and Pep-57 structures (yellow)

An underappreciated, but critical, aspect of structure-based design in the context of peptidic macrocycles is the difficulty in fitting large molecules into X-ray density correctly. The tools available for X-ray crystallography model refinement are better developed for protein modeling than for ligand modeling. Very often, the modeled ligand coordinates yield very high energy values, and modeled coordinates often contain serious errors. This has been established in a number of studies concerned with estimating bound ligand strain energy [15–22] and studies and perspectives involving X-ray model accuracy [12, 13, 23–25]. Figure 5A shows the comparison between the deposited PDB ligand coordinates for Pep-01 (gray carbons) and the re-fit coordinates using the xGen approach [12, 13]. Overall, the ligand had been well-modeled, but one of the chiral centers of the ligand was incorrect (red arrow), causing a distortion to the ring-closing thioether linkage.

Figure 5B shows a much more serious set of problems with the deposited structure of Pep-57 (orange carbons) compared to the xGen re-fit (yellow). Note that Pep-57 differs only at position 7 from the lead compound Pep-01, lacking the N-methyl and replacing Gly with Ser. Three cis-amide bond configurations are highlighted (red arrows), but the structure contains numerous high-strain features. If we consider the overlay of the two deposited peptide variants in Fig. 5C, we would conclude that the macrocyclic backbone took on substantially different conformations despite only two minor differences between the ligands (both at position 7). However, as is clear in Fig. 5D, the two variants adopt nearly identical backbone configurations when correctly fit into the X-ray density of PDB code 6PV9 and 5O4Y.

The importance of the above comparison for the purpose of predictive modeling is that the NMR solution ensemble of Pep-01 and both Pep-01 and Pep-57 bound crystal structures agree extremely closely with respect to their conformations. They are nearly identical for the macrocyclic backbone and for the large, common substituents that make strong contact with PD-L1. High-quality fitting of low-energy conformational ensembles, whether to a set of NMR-determined restraints from a pre-organized solution ensemble or to X-ray density, is required in order to accurately model the likely bound states of analogs.

Results from the scheme presented in Fig. 3 do not vary substantially whether making use of the lowest energy conformer from the NMR ensemble (Fig. 4C) or deriving analogous molecular subfragments from either the 6PV9 or 5O4Y structures. Because an NMR ensemble can be obtained regardless of having a protein target structure, in what follows, all results reflect the conformational restraints that were derived from the experimental NMR data on Pep-01.

**Fig. 6:**
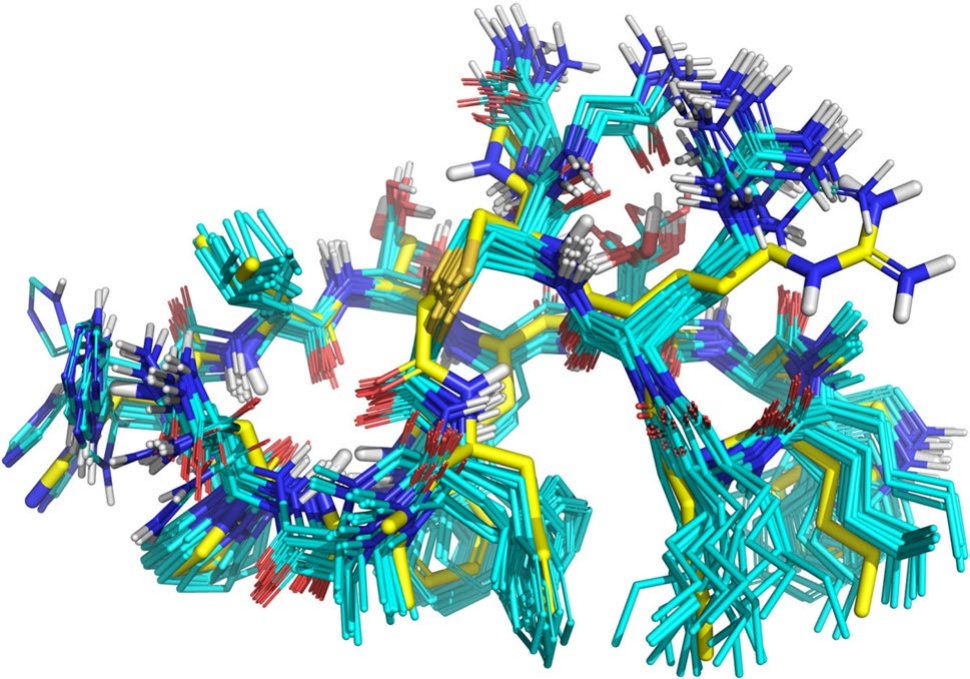
Non-redundant low energy (within 5 kcal/mol of the minimum) pool of conformers of Pep-57 (cyan) superimposed on the crystallographic pose of Pep-57 from 5O4Y (yellow)

Figure 6 shows the low-energy conformational ensemble for Pep-57 superimposed onto its crystallographic pose. The ensemble was derived using the torsional restraints from the lowest energy conformer of Pep-01’s NMR-derived solution ensemble (recall Fig. 4C). The full ensemble contained conformers with 1.0Å RMSD to the bound state and the low energy pool depicted in Fig. 6 contained conformers with 1.4Å RMSD to the bound state. Deviations from the crystallographic pose were in the solvent-facing residues, with very tight correspondence among residues involved in protein binding. The NMR-derived torsional restraints provide an effective means to identify conformers close to the bound states of Pep-01 analogs.

### Relationship of estimated binding enthalpies and experimentally measured binding affinities

**Fig. 7:**
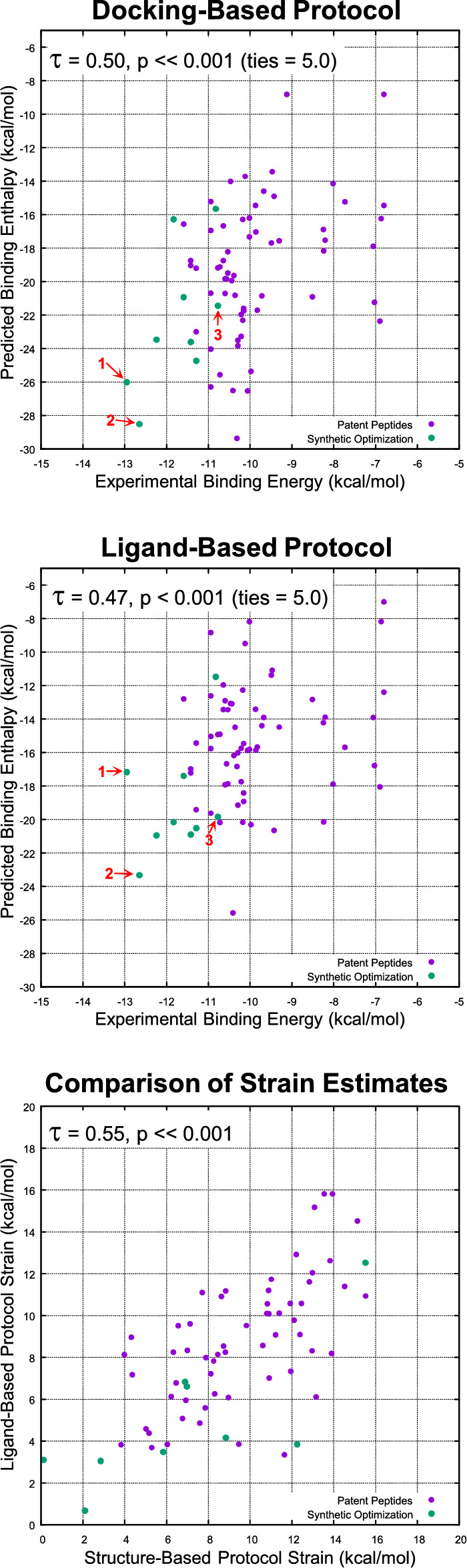
Relationship of predicted binding enthalpy to binding free energy (calculated from experimentally determined IC_50_ values) for the structure-based protocol (top) and ligand-based protocol (middle), and comparison of bound ligand strain estimates between the two protocols (bottom)

Figure 7 (top) shows the relationship between experimental (X-axis) and structure-based-protocol predicted binding for 63 patent peptides (violet) and 9 subsequently made and tested project compounds (green). Note that the assays were slightly different (e.g. for Pep-01, the difference was roughly sixfold, with poorer nominal binding for the HTRF patent assay), but are generally comparable. The points labeled 1, 2, and 3 correspond, respectively, to BMT-174900, BMT-153099, and BMT-139699 from Fig. 1. Kendall’s Tau ($\tau$) was 0.25 (p < 0.001) with ties being counted as exact values, increasing to $\tau = 0.50$ with prediction value ties defined as being within 5.0 kcal/mol of one another (p $\ll$ 0.001). Given two analogs whose predicted binding enthalpy values differed by 5 kcal/mol, the likelihood that they were ranked correctly was 75%. Pearson’s correlation (*r*) was 0.48. Of note, the clinical candidate (BMT-174900) and an analog with similar activity (BMT-153099) were among the best scoring 6 of 72 compounds. The mean pK_d_ of the ten best predicted analogs was 8.1.

Figure 7 (middle) shows the relationship between experimental (X-axis) and ligand-based-protocol predicted binding for 63 patent peptides and nine subsequently made and tested project compounds, with points colored as above. Kendall’s Tau ($\tau$) was 0.25 (p $=$ 0.001) with ties being counted as exact values, increasing to $\tau = 0.47$ with prediction value ties defined as being within 5.0 kcal/mol of one another (p < 0.001). Given two analogs whose predicted binding enthalpy values differed by 5 kcal/mol, the likelihood that they were ranked correctly was 73%. Pearson’s correlation (*r*) was 0.42. Of note, the clinical candidate (BMT-174900) was *not* among the best-scoring compounds in the ligand-based protocol. The ligand-based protocol has a fundamental lack of information regarding the new favorable interactions of BMT-174900 with PD-L1 that are evident in the structure-based protocol. However, five highly active analogs from the lead optimization effort were among the top 11 predictions. The mean pK_d_ of the ten best predicted analogs by the purely ligand-based protocol was 8.0.

The direct correlation between the structure-based and ligand-based strain estimates was high (Fig. 7, bottom, with $\tau = 0.55$, p $\ll$ 0.001, $r = 0.86$, and mean absolute difference being 2.2 kcal/mol). This reflects the degree to which the ligand-based predictions of bound ligand pose matched those from docking (discussed below). Bound ligand strain, by itself, was the major predictive factor of experimentally measured analog activity, which is why the purely ligand-based approach exhibited a similar level of predictive value to the structure-based approach.

### Expectations for ligand strain

It is not clear the extent to which the predictive value of ligand strain is a general property for macrocyclic peptides that result from the type of intensive affinity-based screening used to identify Pep-01 [1], but it is possible to quantify the likelihood that ligand strain can be leveraged in lead optimization. We have recently shown that bound ligand strain follows a size-dependent probability distribution [15]. For Pep-01, the expected bound strain is roughly 24 kcal/mol and the expectation is that 95% of cases will fall between in the range of 14–34 kcal/mol. From the real-space refined coordinates of Pep-01, we obtained an estimated bound strain of 12.7 kcal/mol—clearly very low. From re-docking Pep-01 into its cognate protein structure, an analogous process to that used for the analog compounds, we obtained an even lower strain estimate: 2.3 kcal/mol. Note that because the conformer pool for Pep-01 was derived from the torsional preferences of its own solution-state, it is likely that the strain estimate from docking is systematically lower than that of the analog compounds.

Whether considering the strain estimate from crystallography (very low for its size) or from docking (extremely low), one should expect that many changes to the lead compound’s structure will result in significant increases in strain. So, maintaining low strain in the design process is clearly indicated based on where the lead compound falls within the expected strain distribution. Here, using either the structure-based protocol or the ligand-based protocol, we see that the most active analogs have extremely low strain compared with expectations: an average of 7.6 kcal/mol for those with activity $\ge$ 8.0 pIC_50_ units. Conversely, the least active analogs have approximately double the strain: an average of 14.7 kcal/mol for those with activity $\le$ 6.0 pIC_50_ units (still quite low, but the changes were modest).

Recall Pep-05 from Fig. 3, which was a Phe to Ala change at position 1, resulting in a decrease in activity of nearly 3 log units. The change resulted in a loss of less than 0.5 kcal/mol in intermolecular binding energy compared with Pep-01. However, the bound strain estimate increased by roughly 8 kcal/mol for Pep-05. The predicted loss in activity between Pep-01 and Pep-05 based on intermolecular energy and strain is overestimated, but it correctly deprioritizes Pep-05 as an analog for synthesis and testing. This type of effect is likely to be general. Large, rigid substituents such as phenylalanine create conformational constraint by excluding possible conformational states. Changes that decrease either the size or rigidity of such substituents are likely to reveal different (and lower) global minima relative to the bound conformational energy.

Pep-50 from Fig. 3 is interesting for similar reasons. The deletion of a single methylene from the Pro residue at position 4 makes a small change to the interaction footprint, leading to a decrease in intermolecular binding energy of 0.7 kcal/mol. The impact of the change on estimated strain was larger: an increase of just under 5 kcal/mol. As with Pep-05, the predicted degree of loss in activity was overestimated, but the important aspect is that the rankings of the compounds were correct: Pep-01 > Pep-50 > Pep-05. Further, while the gap between Pep-01 and Pep-05/Pep-50 was overestimated, the gap between Pep-50 and Pep-05 was quite closely predicted ($\Delta \Delta$G of 3.0 kcal/mol predicted vs. 2.7 experimental).

Because of the dominant effect of estimated strain, both the structure-based and ligand-based protocols agreed on the ranking of these compounds.

### Protein structure adds exploitable information

In the structure-based protocol, docking score is defined as the estimated intermolecular binding enthalpy ignoring ligand strain. By itself, the correlation between this score and experimentally determined binding affinity was weak ($\tau = 0.12$, p $=$ 0.07). However, despite strain being the largest explanatory component of analog activity, predicted interactions between analogs and PD-L1, both quantitatively and qualitatively, had significant value.

**Fig. 8:**
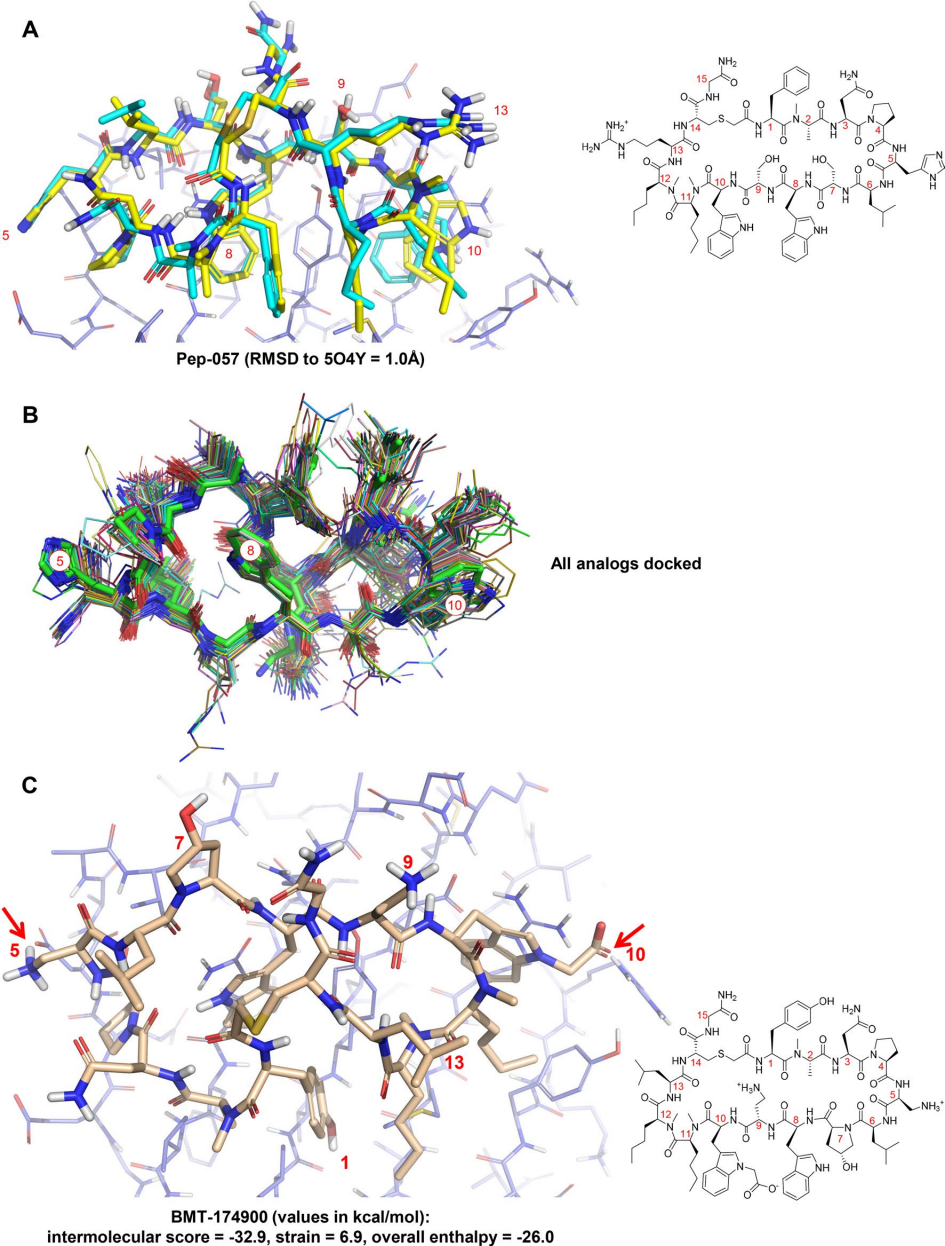
Docking of analogs to PD-L1 (PDB Code 6PV9): **A** comparison of predicted (cyan) and experimentally determined (yellow, PDB Code 5O4Y) bound pose of Pep-57; **B** top-scoring docked poses of all analogs (seen from the protein interface) superimposed on the bound pose of Pep-01 (green carbons); **C** predicted bound pose for BMT-174900

Figure 8A shows the predicted pose of Pep-57 (a variant of Pep-01 differing slightly at position 7) when docked into the cognate protein conformation of Pep-01. The predicted pose (cyan) was just 1.0 Å RMSD from the experimentally determined pose (yellow). Figure 8B shows the top-scoring docked poses for all 72 of the analogs. The torsionally restrained search procedure yielded compliant conformers for all compounds, which exhibited largely congruent binding interactions at the protein interface.

Figure 8C shows the predicted binding mode of BMT-174900, with two salt-bridges to the protein at positions 5 and 10 (marked with red arrows). The structure-based protocol predicted a marked improvement (3.7 kcal/mol in intermolecular score) progressing from Pep-01 to BMT-174900. The strain estimates from the structure-based and ligand-based protocols differed by less than 0.1 kcal/mol. It was the estimate of intermolecular binding enthalpy from the structure-based protocol that led to the much better ranking of BMT-174900 (see the points labeled 1 in the top and middle plots of Fig. 7).

Recall BMT-153099 from Fig. 1, which differs from BMT-174900 only at position 10, with a benzothiophene rather than the substituted indole. In the pure binding assay, the two compounds exhibited very similar activity. BMT-153099 was the only analog with a calculated intermolecular binding score (units of pK_d_) higher than BMT-174900 and the calculated strain estimate was lower in both protocols. Both protocols incorrectly ranked BMT-153099 with respect to BMT-174900, with the structure-based protocol predicting a smaller gap than the ligand-based protocol. The docked pose of BMT-153099 was not significantly different than BMT-174900, with the change in score being driven by the $\pi$-cation interaction of the thiophene compared with the substituted indole.

BMT-139699 differed from BMT-153099 only at position 7, replacing the hydroxyl with a proton. Because the hydroxyl at position 7 is completely solvated, it was unsurprising that the estimated intermolecular binding energy differed only slightly (with BMT-153099 being 0.3 kcal/mol lower in intermolecular energy). Note, however, that the difference in experimentally measured activity corresponded to just under 2 kcal/mol. Here again, the estimated strain pointed in the correct direction, with significantly increased strain estimates for BMT-139699: 6.7 and 3.5 kcal/mol by the structure-based and ligand-based protocols, respectively.

### Comparison of predictions for bound ligand poses

**Fig. 9:**
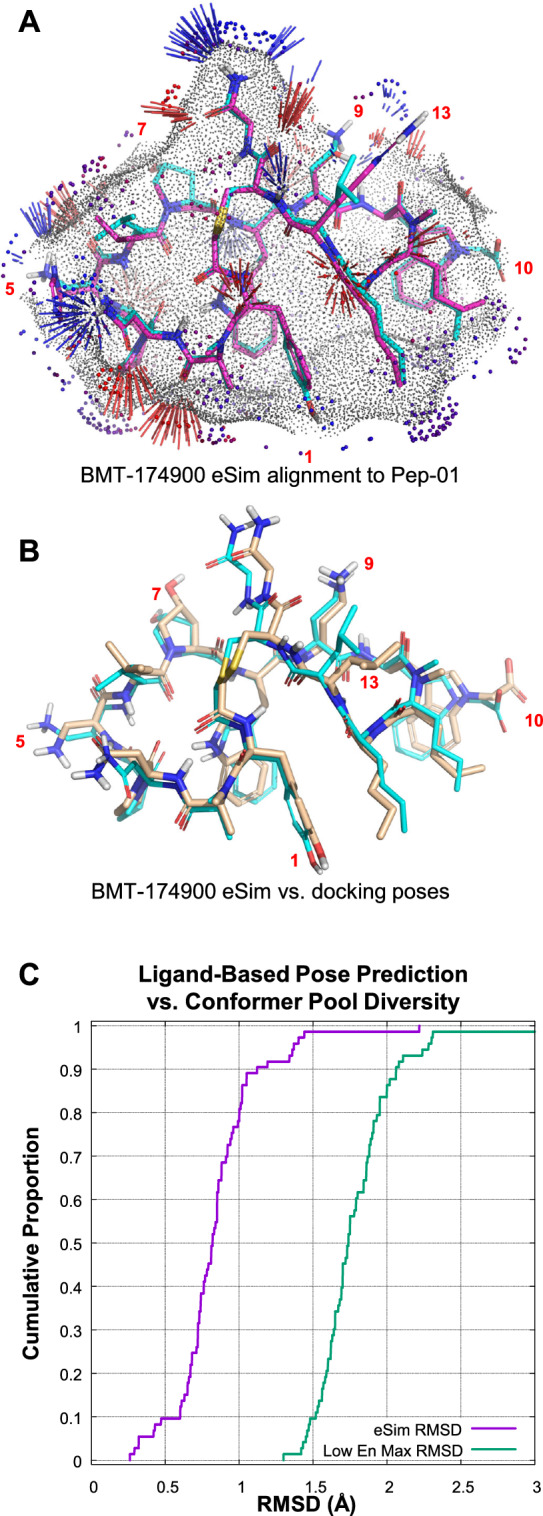
Pose prediction accuracy for the ligand-based protocol: **A** optimal eSim alignment of BMT-174900 to Pep-01; **B** superimposition of pose prediction from ligand similarity (cyan) and docking (tan); and **C** relationship of pose prediction accuracy for the ligand-based protocol (violet) to the poorest possible result from the low-energy pose pool for each analog (green)

The structure-based protocol produced a highly accurate docking for Pep-57 and convincing poses for the remaining analogs (see Fig. 8). The parallel ligand-based protocol also predicted poses for all analogs using the eSim method [14] in order to derive an estimate for bound conformational energy. Figure 9A shows the optimal alignment of BMT-174900 to Pep-01 using ligand-based pose prediction. Gray dots show the parts of the molecular surfaces that are congruent, with notable differences only at positions 5, 10, 9 and 1. Red and blue sticks show congruence of hydrogen bond donors and acceptors, including directionality. Small spheres in the red to blue color spectrum indicate areas where the electrostatic fields of the molecules are congruent.

Figure 9B shows the comparison of the bound pose predictions of BMT-174900 from the structure-based protocol (tan) and the ligand-based protocol, with an RMSD of 0.9 Å. There are slight differences in the poses at positions 5 and 10, where docking identified quantitatively significant interactions with the protein, but where the ligand-based approach simply saw differences between Pep-01 and BMT-174900. Figure 9C shows the cumulative histogram of RMSD for the ligand-based pose predictions compared to the poorest expected RMSD for each analog. The RMSD values for the eSim predictions were derived by comparing the similarity-predicted poses against the top-scoring pose family from docking for each analog. The pessimistic RMSD values were derived by considering the lowest 10 kcal/mol conformers from each analog’s pool and identifying the most deviant conformer compared with the top-scoring pose family from docking.

Over 80% of the cases showed conformer matches to docking of 1.0 Å RMSD or less in the ligand-based protocol, with 98% being under 1.5 Å RMSD. This was not simply because the torsionally restrained pools contained no conformers that deviated from the likely docked poses. The pools contained a diversity of conformations for each analog, typically containing examples deviating 1.5 to − 2.5Å from the docked configurations. The close relationship between the strain estimates for the structure-based and ligand-based protocols stemmed from the quantitative similarity in their predicted bound poses for the analogs.

Note, however, that this was a structure-enabled project, which influenced the design of analogs. In this restrospective analysis, without protein structure, the substitution on the Trp indole at position 10 would probably not have been explored in using a systematic “conservative” strategy. Combinatorial exploration of such diverse sidechain variants would yield an extremely large space of analogs to prioritize. It is conceivable that a position-by-position sequential optimization, essentially an iterative line search strategy, could be used in a “blind” exploration. Such a strategy assumes that the effects of positional variations will be largely additive.

### Computational cost

Large macrocycles present special challenges, particularly regarding the computational complexity of conformational search. The *fgen_deep* search approach requires roughly ten-fold more time than the standard thorough ForceGen search protocol for the compounds studied here. Roughly speaking, using the thorough ForceGen search protocol for all conformational searches, roughly 1000 compounds per day can be run on a 100-node cluster of 36-core nodes, with ten-fold fewer using the deep search protocol. Deeper conformational search produced stronger correlations between estimated enthalpies and experimentally determined activities, but for ranking larger sets of candidate analogs, the faster protocol would be useful for eliminating poor candidates. Given the availability of cloud-based high-performance computing, with schemes that trade perfect availability against cost, the trade-offs between calculation speed and accuracy are complex.

## Conclusion

Systematic optimization of large macrocyclic peptide ligands is a serious challenge. We have described a lead-optimization approach using the PD-1/PD-L1 system as a retrospective example of moving from initial screening hit to clinical candidate, using either a structure-enabled or a purely ligand-based approach. Armed only with data from the NMR solution ensemble of the lead compound from affinity-based selection, significant efficiency can be gained. In this study, roughly 50% of analogs could be eliminated from synthetic consideration without breaking the successful optimization path that resulted in BMT-174900. Protein structural information is clearly beneficial, both in terms of the quantitative value in ranking analogs and in terms of helping to guide the design of specific analogs. With the additional information provided by the structure-based protocol, roughly 80–90% of analogs could be eliminated from synthetic consideration.

A surprising aspect of this study is the central importance of bound ligand strain in making predictions. Essentially, the propensity of each macrocyclic analog to adopt a bound conformation very similar to the lead compound was the largest explanatory component of activity. Relatively small ligand modifications, which may have minimal effects on predicted bound pose or intermolecular interactions, often lead to large changes in estimated strain that have dominating effects on overall binding energy estimates.

In terms of prospective application of the methods described here to macrocyclic peptide lead optimization, a critical factor is whether solution ensembles are pre-organized for binding to the target site in question. Because affinity-based selection of peptides such as those studied here is a relatively new technological approach, it is difficult to predict how likely such pre-organization may be. If a coherent conformational ensemble exists in solution, which can be established through NMR-based biophysical characterization, and the on-rate of association between the ligand and protein is fast, it is reasonable to pursue the ligand-based strategy. Given experimental activity and predicted rank-order over a conservatively chosen set of analogs, the strategy could be quickly validated or rejected.

In the case that X-ray data is available for a bound ligand exemplar, the structure-based protocol could be assessed similarly. In both situations, the extent to which the protein exhibits significant flexibility on binding different analogs is a potentially confounding factor. In the work presented here, PD-L1 does not appear to exhibit much conformational variability from analog to analog. However, there is quite a significant difference between the apo form of PD-L1 and that bound to the peptidic macrocycles studied here. Given the added value of structural guidance, both in terms of improvements in the computational protocol and in terms of aiding the design process, whenever possible, the structure of at least one protein-ligand complex should be sought.

Effective macrocyclic conformational search is critical, whether in the context of NMR-based restraints, X-ray ligand refinement, using partial torsional restraints for docking or ligand similarity calculations, or agnostic search for nominal global minima. Our expectation is that, in many cases, lead optimization for peptidic macrocycles can be made more productive using a multi-disciplinary approach that combines biophysical data with practical and efficient computational methods.

## Methods and data

### Molecular data set

The macrocyclic peptides studied here included 64 from the original patent disclosure [2], each of which had an associated IC_50_ for inhibition of HTRF-based PD-L1/PD-1 binding. Also included were 9 compounds from various time-points during project lead-optimization, each of which had an associated IC_50_ measured for in a HEK293 cell-based assay in which PD-L1 is recombinantly overexpressed on the cell surface and inhibition of binding to soluble recombinant PD-1 is measured.

### Experimental NMR data for Pep-01

#### NMR sample preparation of Pep-01

A 5.1 mg sample of Pep-01 was dissolved in a 0.65 mL binary mixture of 30% perdeuterated acetonitrile + 70% glycine buffer in 100% H_2_O (30 mM glycine-*d5*, pH = 2.5) and placed in a 5 mm thin-wall tube (Wilmad precision NMR tube: 541-pp-7–5).

#### NMR data acquisition

All NMR spectra were acquired on an AVANCE NEO spectrometer operating at 700.14 MHz equipped with a TCI 5 mm cryoprobe and TopSpin version 4.1.3. Spectra acquired at 15 °C:

- Proton 1D with Excitation-Sculpting water peak suppression [26], water resonance frequency and spectrum center at 4.585 ppm.
- ^1^H-^13^C HSQC with DEPT-editing [27], sw = 14.88 ppm, sw1 = 200 ppm, o1p = 4.589 (on-resonance with water peak), o2p = 90 ppm, td = 4096, td1 = 1024, relaxation delay d1 = 2.5 s, echo/anti-echo acquisition.
- ^1^H-^15^N HMQC with Watergate water peak suppression [28], sw = 14.88 ppm, sw1 = 40 ppm, o1p = 4.589 (on-resonance with water peak), o3p = 116 ppm, td = 4096, td1 = 256, d1 = 1.5, relaxation delay d1 = 1.5s, STATES-TPPI acquisition.
- ^1^H-^13^C HMBC [29], sw = 14.88 ppm, sw1 = 200 ppm, o1p = 4.589, o2p = 95 ppm, td = 4096, td1 = 512, echo/anti-echo acquisition, d1 = 2.0 water peak suppression by a combination of on-resonance pre-saturation with a saturation field of 50 Hz amplitude plus a 2 ms soft water flip-pack pulse preceding the last proton echo pulse (see supplemental material for additional details), long-range coupling delay was set to 1/2 * 8Hz. The spectrum was processed in magnitude mode in F2 and phase-sensitive mode in F1.
- ^1^H-^1^H TOCSY [30], sw = 14.88 ppm, sw1 = 14.28, td = 4096, td1 = 400, relaxation delay d1 = 2.0 s, mixing time = 0.075 s, water peak suppression by CW-presaturation and Excitation Sculpting, suppression of peak shape distortion by inclusion of a zero-quantum filter [31], STATES-TPPI acquisition mode.
- Double-quantum filtered-COSY [32] with Excitation Sculpting water-peak suppression [26]—see supplemental materials for details on pulse sequence customization, the sw = 14.88 ppm, sw1 = 14.28, td = 4096, td1 = 2048, d1 = 2.0 s.
- 2D-NOESY: sw = 14.88 ppm, sw1 = 14.28, td = 4096, td1 = 2048, d1 = 3.5 s, CW-water peak pre-saturation during the relaxation delay and the mixing interval of 0.5s.

Spectra acquired at 283 °K:

- 2D-NOESY: sw =14.88 ppm, sw1 = 14.28, td = 4096, td1 = 800, d1 = 3.5s, water peak suppression by Excitation Sculpting, mixing times; 0.1, 0.2, 0.3, 0.4, 0.5s
- ^1^H-^13^C HSQC, ^1^H-^15^N HMBC, ^1^H-^1^H TOCSY employing parameters as depicted in list of experiment which were recorded at 288 K.
- T1-measurements: Bruker inversion recovery pulse program, inversion recovery delays: 0.001, 0.4, 1.0, 2.5, 5.0, 9.8 s, d1 = 10 s, water peak suppression using CW-presaturation of a 50 Hz rf field. Data analysis in the TopSpin dynamics module. Processing in TopSpin T1/T2-relaxation module

#### NMR-resonance assignments

Resonance assignments were performed on the 288K dataset using 1D-proton, DQF-COSY, ^1^H-^1^H TOCSY, 2D-NOESY (d8=500ms), ^1^H-^13^C HSQC, ^1^H-^13^C, HMBC, and ^1^H-^15^N-HMQC using ACD/Lab workbook version 2020.2.0 (Advanced Chemistry Development Inc., Toronto, Ontario, Canada). All ^13^C chemical shifts were within theoretical limits of the built-in chemical shift prediction module (add citation of ACD/Labs). Resonance assignments were mapped to the 283K dataset using ACD labs and peak lists were exported. The peak lists were subsequently imported into Sparky [33] where cross-peaks were manually picked in the 200 ms mix time NOESY spectrum. Peak volumes were generated by the sum-over ellipse method. Proton shift assignments and NOESY peak list were exported in XEASY-format [34]. A total of 393 peaks across both sides of the diagonal were picked.

Torsion angle restraints were derived using the modified Karplus equation [35]. The J_HN-Hα_ values were extracted from 1D spectra with minimal apodization. Amides with J_HN-Hα_ > 8.0 Hz, HIS5, LEU6, TRP8, SER9 and ARG13 were assigned Phi angles from − 155 to − 95.

NMR-peak assignments and computation of initial 3D-structures:

CYANA [36] structure calculation required the definition of unnatural amino acid types in the CYANA-library format. CYANA library files of unnatural amino acid types were generated by CYLIB [37]. Editing of CYLIB-generated CYANA-library files was aided by atom number to name conversion utility in CYANA. Separate upper bound (*.upl) and lower bound (*.lol) files were generated to link the sulfur dummy atom, and the residue PHS1 $\Omega$ angle was set to 160–200 to properly assign the disulfide geometry for the ring closure. Cis peptide bonds were set in the CYANA *.seq file for residues 2 and 11 based on observation of cis NOE patterns including, H$\alpha$–H$\alpha$ NOEs for residues 10–11 and strong N-methyl to aromatic NOEs between residues 2 to 1.

Peak integral to upper distance bounds restraints were generated by CYANA [36] using the built in “noeassign” command. A total of 165 peaks were assigned and translated into upper-bound distance restraints. Tabulation of short range and long-range NOEs used in the 3D structure calculation are included in the supplemental experimental NMR data. Initial CYANA structures produced an ensemble of 20 3D-structures with average heavy atom RMSD to mean of 0.95 Å (± 0.22 Å). The Ramachandran plot analysis indicated that 61.4% of Phi and Psi angles resided in the most favored regions with an additional 36.8% in the additionally allowed region.

The experimental data yielded 50 distance restraints between single proton pairs, 115 distance restraints where one/both ends contained chemically equivalent protons, and 6 torsional restraints consisting of 1 omega and 5 psi angles. Structure generation and conformational search was done using the deep ForceGen search procedure, described below.

### NMR restraints with chemically equivalent protons

Given that over two-thirds of the restraints that affect conformational search for Pep-01 involved chemically equivalent protons, the precise treatment of those restraints was important. It has been argued that the best treatment of equivalent or nonstereoassigned protons in calculations of biomacromolecular structures is done by considering the so-called $r^{-6}$ averaged distance [38, 39]. A common alternative approach is to make use of the so-called center-averaged distance (along with a pseudo-atom correction to the restraint distance). Here, we introduce a new alternative, which closely approximates the $r^{-6}$ averaged distance, but which is simpler and more efficient to calculate. The following defines three different distances between equivalent spin groups *a* and *b*:

1 $$\begin{aligned} r_{eff}&= \left( \frac{1}{n_an_b} \sum _{i,j}r^{-6}_{a_i b_j} \right) ^{-1/6} \end{aligned}$$

2 $$\begin{aligned} a_{cen}&= \frac{1}{n_a} \sum _{i}{a_i} , b_{cen} = \frac{1}{n_b} \sum _{j}{b_j} \end{aligned}$$

3 $$\begin{aligned} r_{cen}&= r_{a_{cen} b_{cen}} + \delta _{ab} \end{aligned}$$

4 $$\begin{aligned} r_{qmin}&= \min _{i,j}(r_{a_i b_j}) \end{aligned}$$

where $a_i$ and $\delta _{ab}$ is the pseudo-atom correction for the two spin groups. Equation 1 defines the $r^{-6}$ average distance, which requires calculation of $n_a n_b$ distances, a sum over the inverse of their sixth power, and finally the normalized inverse of the sum’s sixth root. Equations 2, 3 define the center-averaging distance, which requires two centroids, their distance and addition of a correction term. Equation 4 defines the “qmin” distance, which requires computing the minimum over $n_a n_b$ distances (or the square root of the minimum over $n_a n_b$ squared distances, which is more computationally efficient). Further, the first derivative of *qmin* distance depends only on the two atomic locations that gave rise to the minimum distance.

**Fig. 10:**
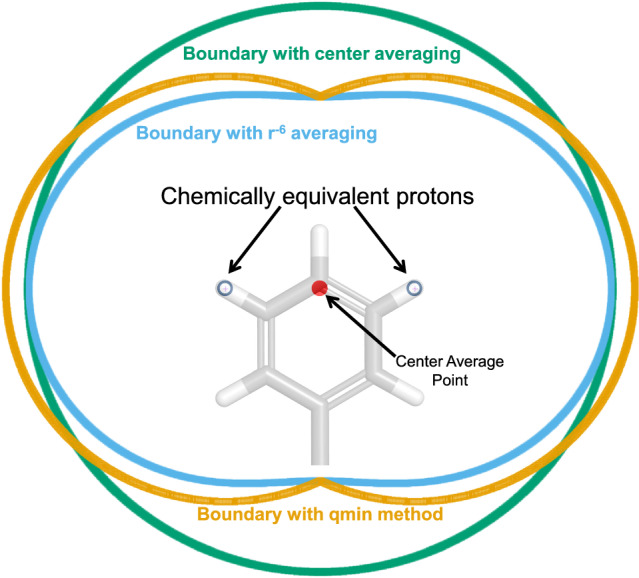
Illustration of the “qmin” approach to restraint enforcement compared with center-averaging or $r^{-6}$ averaging

Figure 10 shows the comparison of the restraint boundary in two dimensions for a single proton to the two chemically equivalent protons on a phenyl group. The *qmin* method closely follows the $r^{-6}$ averaged boundary, with the center-averaging approach (with a well-chosen pseudo-atom correction) deviating significantly. Given the simplicity of implementation, computational efficiency, and relatively small deviation from the $r^{-6}$ average, the *qmin* approach is appropriate in cases where a calculation requiring a restraint on chemically equivalent protons falls in the inner-loop of a complex optimization procedure.

### Deep ForceGen search

The ForceGen conformational search method has been previously described [8, 9]. For small, drug-like molecules, the *-pquant* level of conformational elaboration is likely to be sufficient to make accurate estimates of global minima in the vast majority of cases, based on the roughly 98% success rate of identifying close-to-crystallographic conformers ($\le$ 1.5 Å RMSD) beginning from random starting conformations [9]. However, particularly for large, peptidic macrocycles, we have developed an iterative approach to conformational search in order to better ensure adequate sampling [13]. This iterative search has been implemented as a command within the Tools Module of the Surflex Platform, called *fgen_deep*.

Beginning from a single input conformer, the *fgen_deep* procedure performs a standard ForceGen search, with the resulting conformer pool being clustered by RMSD. If the resulting N lowest-energy clusters contain new conformations compared with prior rounds, search is iterated beginning with the lowest energy conformers from each of the N new clusters. Multiple rounds of this are carried out, each time consolidating the full set of conformers into a non-redundant set within a specified energetic window prior to clustering. The process is iterated until no new low-energy clusters are identified.

**Fig. 11:**
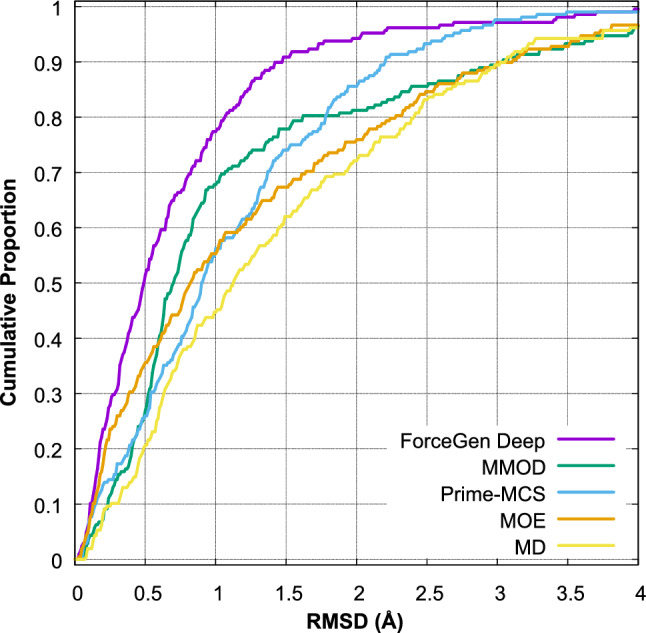
Performance of the deep ForceGen search methodology on the Prime MCS 208 example macrocycle benchmark

Figure 11 shows the performance of the *fgen_deep* procedure on a benchmark of 208 macrocycles [7, 9], compared with low-mode MD implemented within MOE and MacroModel [4, 6] and with the Prime MCS approach and pure MD simulation [7, 9]. We see that, at the 1.5 Å RMSD threshold, a success rate of just over 90% was achieved using the *fgen_deep* approach. Note that this still falls short of the 98% seen for “normal” small molecules, but it is substantially better than the alternative methods, whose success rate ranged from 62 to 78%.

### Computational procedures

A rough outline of the computational protocols is provided here (see Data and Software Availability for full details). Following is the NMR-restrained conformational search of Pep-01:
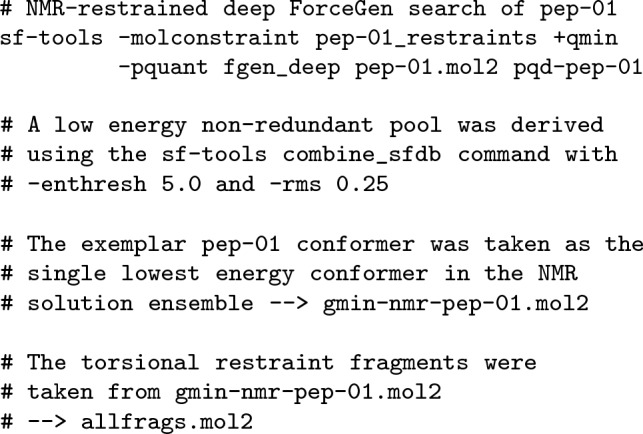


Following is an outline of the structure-based and ligand-based protocols that make use of the torsional restraint fragments derived above from the NMR solution ensemble of Pep-01:
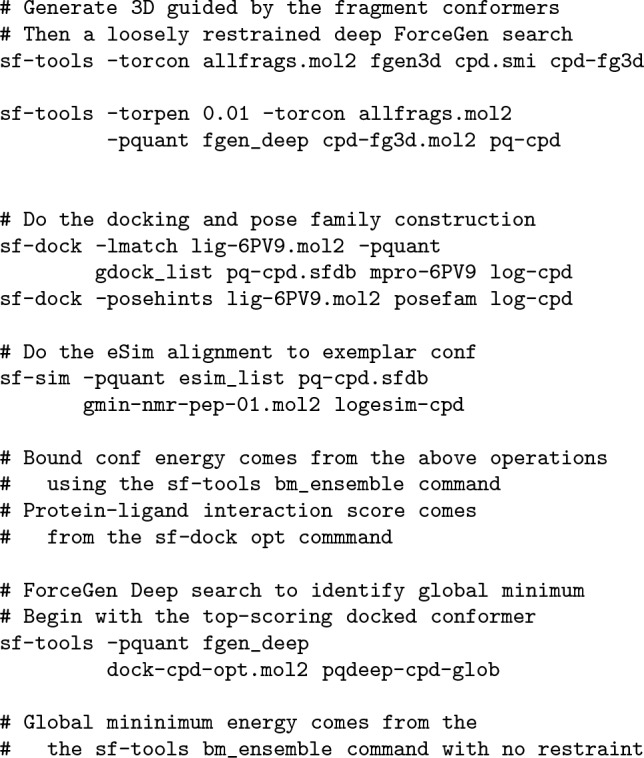


For all conformational search (NMR restrained or agnostic), real-space ligand refinement, docking, ligand-similarity calculations and related strain estimates, we employed version 5.1 of the Surflex Platform (BioPharmics LLC, Sonoma County, CA 95404).

## Data Availability

A freely downloadable data archive containing additional computational and experimental details is available at http://jainlab.org/downloads. The archive contains supplementary details regarding the NMR experimental data and a summary spreadsheet of ligand structures and activity values along with calculated global minimum energy values, bound energy values, derived strain estimates, and intermolecular binding energy values. The archive also contains scripts to reproduce the major results of the paper along with SMILES-format input structures, generated 3D ligand structures, conformational ensembles, protein structures, and NMR restraint data. All software employed herein is commercially available.
