# Correction: Complex peptide macrocycle optimization: combining NMR restraints with conformational analysis to guide structure-based and ligand-based design

**DOI:** 10.1007/s10822-024-00556-2

**Published:** 2024-03-13

**Authors:** Ajay N. Jain, Alexander C. Brueckner, Christine Jorge, Ann E. Cleves, Purnima Khandelwal, Janet Caceres Cortes, Luciano Mueller

**Affiliations:** 1 Research and Development, BioPharmics LLC, Sonoma County, CA USA; 2 Bristol-Myers Squibb Company, Princeton, NJ USA

**Correction to: Journal of Computer-Aided Molecular Design (2023) 37:519–535** 10.1007/s10822-023-00524-2

In the original publication, the Fig. 3 was cropped during production and published incorrectly. The corrected Fig. 3 should have appeared as shown below.

**Fig. 3:**
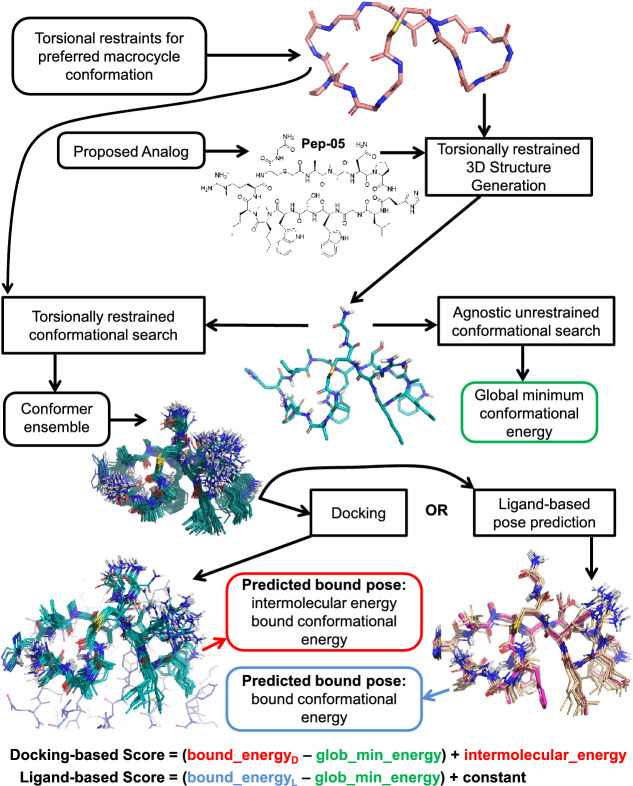
Scheme for exploiting a macrocycle conformational preference to predict a bound pose, either using docking (protein structure shown in slate carbons at bottom left) or ligand similarity (exemplar conformer target shown in magenta carbons at bottom right). For the ligand-based score, a constant value of − 24.0 kcal/mol was added to the estimated strain energy in order to put the scores from the two protocols on the same rough scale

The original article has been corrected.

